# Pneumococcal vertebral osteomyelitis at three teaching hospitals in Japan, 2003–2011: analysis of 14 cases and a review of the literature

**DOI:** 10.1186/1471-2334-13-525

**Published:** 2013-11-08

**Authors:** Hiromichi Suzuki, Daisuke Shichi, Yasuharu Tokuda, Hiroichi Ishikawa, Tetsuhiro Maeno, Hidenori Nakamura

**Affiliations:** 1 Department of Clinical Laboratory Medicine, Tsukuba Medical Center Hospital, 1-3-1 Amakubo, Tsukuba 305-8558, Japan; 2 Graduate School of Comprehensive Human Sciences, University of Tsukuba, 1-1-1 Tennoudai, Tsukuba 305-8575, Japan; 3 Department of Infectious Diseases and Rheumatology, Seirei Mikatahara General Hospital, 3453 Mikatahara, Kita-ku, Hamamatsu 433-8558, Japan; 4 Department of Medicine, Mito Kyodo General Hospital, University of Tsukuba, 3-2-7 Miyamachi, Mito 310-0015, Japan; 5 Department of Respiratory Medicine, Tsukuba Medical Center Hospital, 1-3-1 Amakubo, Tsukuba 305-8558, Japan; 6 Department of Respiratory Medicine, Seirei Hamamatsu General Hospital, 2-12-12 Sumiyoshi, Naka-ku, Hamamatsu 430-8558, Japan

**Keywords:** Pneumococcal infections, Spinal infections, Spondylodiscitis, *Streptococcus pneumoniae*, Vertebral osteomyelitis

## Abstract

**Background:**

Pneumococcal vertebral osteomyelitis (PVO) is a rare disease whose clinical characteristics have not been clarified. This study aimed to investigate the clinical features and outcomes of patients with PVO.

**Methods:**

We retrospectively evaluated all adult patients diagnosed with PVO at three teaching hospitals in Japan from January 2003 to December 2011. All cases were identified through a review of the medical records of patients with invasive pneumococcal disease (IPD).

**Results:**

Among 208 patients with IPD, we identified 14 with PVO (6.4%; 95% CI, 3.5–10%). All 14 patients (nine male, five female; median age 69 years) had acquired PVO outside the hospital and had no recent history of an invasive procedure or back injury. Five patients (36%) had diabetes mellitus, and four (29%) had heavy alcohol intake. Fever (n = 13; 93%) or back pain/neck pain (n = 12; 86%) were present in most patients. The lumbar spine was affected in nine patients (64%) but the cervical spine was the site of infection in four patients (29%). All patients except one had a positive blood culture for *Streptococcus pneumoniae*, and there were no distant infected sites in most patients (n = 10; 71%). Intravenous beta-lactam therapy was initiated within 1 week after the onset of symptoms in 11 patients (79%). No patients died within 30 days, but one patient died from aspiration pneumonia on day 37 after admission.

**Conclusions:**

PVO was relatively common among adult patients with IPD, and mortality was low in this study. *S. pneumoniae* may be the causative pathogen of vertebral osteomyelitis, especially among community-onset cases without a history of invasive procedures or back injury.

## Background

*Streptococcus pneumoniae* is an important pathogen that is the main cause of community-acquired pneumonia [1], meningitis [2], sinusitis [3] and otitis media [4]. *S. pneumoniae* was also the third most common pathogen in bacterial arthritis, identified in 6% of cases [5].

Pneumococcal vertebral osteomyelitis (PVO) is a very rare disease, and the incidence has not been described in previous vertebral osteomyelitis studies [6-8]. In a literature review of 28 cases with pneumococcal spinal infections, Turner et al. [9] reported that most of the patients had symptoms for weeks before diagnosis and that the lumbar spine was the most common site of infection. Severe complications were relatively frequent (endocarditis 4/28, 14%; meningitis 5/28, 18%), and mortality was high (7/28, 25%) [9]. However, these epidemiological data were derived from case reports, which included cases published more than 50 years ago. Currently, there are few clinical studies of PVO available because of the rare incidence of the infection.

In this study, we set out to determine the clinical epidemiology of patients with PVO in a 9-year retrospective review of cases of invasive pneumococcal disease (IPD) at three teaching hospitals with tertiary emergency medical centers in Japan.

## Methods

### Patients

All adult patients with IPD who were admitted to Seirei Mikatahara General Hospital (SMGH, 934 beds), Seirei Hamamatsu General Hospital (SHGH, 744 beds), and Tsukuba Medical Center Hospital (TMCH, 409 beds) from January 2003 to December 2011 were retrospectively reviewed. Patients with IPD were identified from a review of the microbiological records of *S. pneumoniae* detection in samples of blood, cerebrospinal fluid, joint fluid, or pus from the abscess obtained by aseptic techniques. The exclusion criteria were: (1) age ≤17 years; (2) a previous history of IPD; (3) concurrent detection of other pathogenic bacteria from aseptic sites; and (4) patient refusal, as written in the medical chart, to provide personal information for out-of-care purposes. PVO was identified if the blood culture or the pus aspirated from the intervertebral disk space, or paravertebral, epidural or psoas abscesses was positive for *S. pneumonia*. Vertebral osteomyelitis was confirmed by magnetic resonance imaging (MRI) or equivalent imaging methods in the radiologist’s official report [10].

### Data collection

Medical records, nursing databases, and microbiological records from each hospital were reviewed by physicians qualified as Fellows of the Japanese Society of Internal Medicine, and all decisions were based on consensus.

The demographic and background data extracted from patient records included the following: age; sex; body weight; area of residence; functional capacity according to activities of daily living (ADL) before onset of pneumococcal bacteremia (Katz index [11]); comorbidities and the Charlson comorbidity index [12]; use of immunosuppressants or chemotherapy; administration of antimicrobial agents before obtaining samples for blood culture; extent of tobacco and alcohol intake; seasonality; presence of fever, neck or back pain; time from onset of symptoms until treatment; and preceding history of trauma, skin infections or respiratory symptoms. Patients receiving immunosuppressants were defined as those receiving more than 1 mg of prednisolone on a daily basis or any other immunosuppressive agent such as azathioprine or methotrexate. Patients with heavy alcohol intake were defined as those who consumed more than 80 g of ethanol daily. The duration of alcohol intake was not considered in this study.

Clinical and laboratory data included clinical severity, antimicrobial susceptibilities determined by broth microdilution methods, location of infection sites, complications and other sites of infection, initial antimicrobial agents used, total duration of antibiotic therapy, invasive procedures performed, morbidity and mortality. Clinical severity was classified into sepsis and septic shock based on recently described criteria [13]. Urine output and lactic acid concentrations were not evaluated as criteria for severe sepsis. The prothrombin time international normalized ratio (PT-INR) was considered to be ≤1.5 in cases in which prothrombin time was not determined. Other infective sites including meningitis and infective endocarditis were evaluated based on guidelines or established criteria [14,15]. Pneumonia was considered to be present if a blood culture was positive for *S. pneumoniae* and a chest X-ray or computed tomography showed infiltration consistent with pneumonia in the lung field. Other infective sources such as septic arthritis, liver abscess, or retropharyngeal abscess were considered to be other sites of infections when contrast-enhanced computed tomography or MRI showed compatible findings in an official radiologist’s report.

Outcomes were assessed by morbidity and mortality. Morbidity was defined by the presence of any disorder reducing ADL capacity (Katz Index) at discharge compared with ADL before admission. Mortality was evaluated at 14 and 30 days.

### Statistical analysis

We compared the clinical characteristics of patients with PVO with those of patients with other IPD. Categorical variables were analyzed by the χ^2^ test or Fisher’s exact test as appropriate. Continuous variables were compared using the Student *t* test. The SPSS software package (version 20, IBM, Armonk, NY, USA) was used for all analyses. Statistical significance was defined as a two-tailed *p* value <0.05. For the detection rate of PVO among IPD, we computed exact confidence intervals for a proportion from the binomial distributions.

### Ethical considerations

This study was approved by the ethics committees of Seirei Mikatahara General Hospital, Seirei Hamamatsu General Hospital and Tsukuba Medical Center Hospital.

## Results

We identified 219 adult IPD patients during the study period, of whom 11 met the exclusion criteria (seven declined non-medical use of personal information, three had concurrent detection of other pathogenic bacteria, and one was a recurrent case). Therefore, data from 208 cases were reviewed for infective sources. This resulted in identification of 14 patients (6.4%; 95% CI, 3.5–10%) with PVO, followed by 129 with pneumonia or empyema, 25 with primary bacteremia, 23 with meningitis, seven with joint infections and 10 with other infections.

### Clinical characteristics and outcomes

The characteristics and demographic data of the 14 patients with PVO are summarized in Table 1. The median age was 69 years (range, 35–88 years) and five (36%) were women. Compared with patients with other IPDs, heavy alcohol intake was significantly more prevalent in patients with PVO (29% vs. 6%, P = 0.02). Diabetes mellitus was also common among patients with PVO (36%), although its prevalence was not significantly greater than that in IPD cases (19%). No patient was infected with HIV, or had a history of skin infections, invasive procedures or trauma. Only one patient had respiratory symptoms complicated by pneumonia and no patient presented with influenza. While cases of other IPDs were relatively frequent during the winter season (between December and March), this trend was not seen in the PVO group (50% vs. 14% for winter occurrences, P = 0.01).

**Table 1:** Demographic data for cases of pneumococcal vertebral osteomyelitis and comparison with other invasive pneumococcal disease

	**Pneumococcal vertebral osteomyelitis**	**Other invasive pneumococcal disease**	**P-value**
	n = 14 (*)	n = 194 (**)	(*) vs (**)
Age (y) (median)	69 (35–88)	73 (28–95)	*0.433*
Female	5 (35.7%)	70 (36.1%)	*0.999*
Body weight (kg) (median)	52.8 (27.5–80.0)	49.8 (29.5–92.4)	*0.272*
ADL impairment (Katz Index)	0	24/179 (13.4%)	*0.325*
Residents in long-term care facilities	0	15 (7.7%)	*0.605*
Charlson comorbidity index (median)	1 (0–6)	1 (0–9)	*0.461*
Diabetes mellitus	5 (35.7%)	37 (19.1%)	*0.164*
Malignancy	2 (14.3%)	35 (18.0%)	*0.999*
Dialysis	0	5 (2.6%)	*0.999*
Splenectomy	0	3 (1.5%)	*0.999*
Immunosuppressants or chemotherapy	2 (14.3%)	29 (14.9%)	*0.999*
Antibiotics used before culture obtained	1 (7.1%)	28 (14.4%)	*0.698*
Smoking	6/12 (50.0%)	98/181 (54.1%)	*0.780*
Heavy alcohol intake	4 (28.6%)	12 (6.2%)	*0.015*
Seasonality (winter: December-March)	2 (14.3%)	97 (50.0%)	*0.011*
Hospital-onset infections	0	22 (11.3%)	*0.371*
Clinical Severity Scale			*0.224*
Severe sepsis	7 (50.0%)	69 (35.6%)	
Septic shock	0	31 (16.0%)	
Initial antimicrobial therapy			*0.292*
Beta-lactam agents monotherapy	7 (50.0%)	129/189 (68.3%)	
Beta-lactam agents combination therapy	7 (50.0%)	51/189 (27.0%)	
Other antibiotics	0	9/189 (4.7%)	
Duration of antibiotic therapy (days)	52 (20–330)	15 (0–105)	*0.035*
14-day mortality	0/14 (0%)	29/188 (15.4%)	*0.228*
30-day mortality	0/14 (0%)^a^	37/182 (20.3%)	*0.076*

ADL: activities of daily living.

(a) One patient died of aspiration pneumonia during hospitalization on day 37.

Regarding clinical symptoms, fever was noted in 13 patients (93%) and a history of neck or back pain was noted in 12 patients (86%). Two patients without neck or back pain were identified as possible PVO cases by computed tomography before systemic investigation of pneumococcal bacteremia, and the diagnosis was later confirmed by MRI. Most patients (11/14; 79%) received effective antimicrobial therapy within 7 days after onset of suspected symptoms. Only one patient was diagnosed at 1 month after onset, and this patient subsequently developed endocarditis and required an operation.

All patients were hospitalized for treatment and seven patients (50%) presented with severe sepsis, but no patient developed septic shock (Table 1). Drug susceptibility to penicillin G (minimum inhibitory concentration (MIC) ≤ 2 μg/mL) and levofloxacin (MIC ≤ 2 μg/mL) was preserved in all strains.

The lumbar spine (n = 9, 64%) was the most commonly infected site and half of the patients with PVO had an epidural abscess detected by MRI (Table 2). Two patients had other joint infections and one patient had an infection in the sternum. Other sources of infection (each n = 1) were infective endocarditis, liver abscess and pneumonia, retropharyngeal abscess, and meningitis. The diagnosis of most patients (n = 13) was based on the presence of pneumococcal infection by blood cultures, while the diagnosis of one patient was based on the culture of pus obtained from the iliopsoas abscess.

**Table 2:** Infective sites and complications of pneumococcal vertebral osteomyelitis

	**n = 14**	**(%)**
Sites of involvement^a^		
Cervical	4	29%
Thoracic	1	7%
Lumbar	9	64%
Sacral	3	21%
Complications		
Epidural abscess + Iliopsoas abscess	5	36%
Epidural abscess	2	14%
Iliopsoas abscess	3	21%
Infective sites other than vertebral osteomyelitis		
Arthritis^b^	2	14%
Others^c^	4	29%

^a^Three patients had multifocal infection sites.

^b^One patient had arthritis of the left shoulder joint and one had arthritis of the left sacroiliac joint.

^c^Other sites of infection (each n = 1) included infective endocarditis, liver abscess and pneumonia, retropharyngeal abscess, sinusitis, and meningitis. One patient had both pneumonia and liver abscess along with vertebral osteomyelitis.

All patients were treated with β-lactam therapy. Two patients (14%) underwent invasive procedures, including a patient with infective endocarditis who was referred to another acute care hospital for surgery and survived. While 20% (37/182) of patients with other IPDs died within 30 days, none of the patients with PVO died within 30 days. In the patients with PVO, one patient died of aspiration pneumonia on day 37 after admission and three of the survivors (23%) had morbidity at discharge.

## Discussion

We evaluated 14 cases of PVO, which is currently the largest number of patients analyzed in a study of PVO. While diabetes mellitus and heavy alcohol intake were common among patients with PVO, there were no patients with nosocomial *S. pneumoniae* infections, preceding invasive procedures or injuries. Fever with back pain or neck pain was present in most patients and the lumbar spine was the most frequently affected site of pneumococcal infections. Initial treatments were administered in most patients within a week after onset of symptoms and only one patient died during hospitalization.

We summarized adult PVO cases previously published in the last 30 years (n = 26) [9,16-33] in Additional file 1: Table S1. Compared with the previous 26 cases of PVO, the clinical outcome was favorable in our current cases. The better prognosis might have been derived from earlier diagnosis of PVO and an adequate period of antimicrobial treatment in our current cases. The initiation of treatment seemed delayed for weeks after the onset of back pain in previous cases. However it may also be a result of other factors, such as the serotype, and the invasiveness of *S. pneumoniae*. In our current cases and the previous 26 cases, diabetes mellitus and heavy alcohol intake were common comorbidities and most cases also had a history of fever, back pain or neck pain. The lumbar spine was the most frequently affected site and the characteristics were consistent with bacterial vertebral osteomyelitis caused by other pathogens, mainly *Staphylococcus aureus*[6-8,34,35]. Meanwhile, there were some differences in PVO from other vertebral osteomyelitis. First, most of the pneumococcal infections occurred outside hospital in both our study and the previous 26 cases; nosocomial acquisition, preceding operative procedures, skin infections and back injury were rarely described. In addition, preceding or concurrent infections of pneumococcal meningitis were reported in one patient (7%) in our study and in seven patients (27%) from previous cases reports.

In most previous studies of pneumococcal bacteremia or IPD, the incidence of PVO was not described [36-39]. In a few other studies, the incidence was described as 0.2–1.3% [40-42]. Meanwhile, Turner et al. suggested the possible underestimation of pneumococcal spinal infections in a report of eight cases over 13 years of experience at a single university in the United Kingdom [9]. This suggestion is supported by our results. Our study indicates that we should be vigilant for a history of neck or back pain in cases of pneumococcal bacteremia or other pneumococcal infections. Blood cultures should be obtained for patients with fever and also neck or back pain. The possibility of concurrent PVO should be considered in cases of pneumococcal meningitis so that patients receive an appropriate length of antimicrobial treatment. *S. pneumoniae* may be the causative pathogen in vertebral osteomyelitis, especially among community-onset cases with no history of invasive procedures or back injury.

There are several limitations to this study. It was retrospectively conducted at three teaching hospitals in two regions of Japan. The serotypes of *S. pneumoniae* were not evaluated and the pneumococcal vaccination status was not recorded or described in most patients and thus our results might not be the same as those for PVO in other regions. Finally, the investigations for vertebral osteomyelitis were not performed for all patients with IPD and patients in critical conditions could not tolerate such an examination. Thus actual incidence and mortality of PVO might have been higher than that reported here. Larger clinical studies are required, especially to determine risk factors and to formulate appropriate prevention and treatment strategies for PVO.

## Conclusions

Our study suggests that vertebral osteomyelitis may be a relatively common infective site among adult patients with IPD. The mortality in those with PVO could be low with early diagnosis and appropriate management. *S. pneumoniae* should be considered as the important causative pathogen in vertebral osteomyelitis, especially among community-onset cases without a history of invasive procedures or back injury.

## Competing interests

The authors declare no conflicts of interest.

## Authors’ contributions

HS designed the study and participated in collecting the clinical data, data analysis and drafting of the manuscript. YT designed the study and was involved in data analysis and drafting of the manuscript. DS, HI and HN designed the study. They were also involved in supporting the data collection and helping to draft the manuscript. TM designed the study and helped draft the manuscript. All authors reviewed and approved the final manuscript.

## Pre-publication history

The pre-publication history for this paper can be accessed here:

http://www.biomedcentral.com/1471-2334/13/525/prepub

## Supplementary Material

Additional file 1: Table S1 Summary of previously reported adult pneumococcal vertebral osteomyelitis cases (1986–2012). Click here for file
